# Valenced Cues and Contexts Have Different Effects on Event-Based Prospective Memory

**DOI:** 10.1371/journal.pone.0116953

**Published:** 2015-02-03

**Authors:** Peter Graf, Martin Yu

**Affiliations:** Department of Psychology, University of British Columbia, Vancouver, British Columbia, Canada; Mount Royal University, CANADA

## Abstract

This study examined the separate influence and joint influences on event-based prospective memory task performance due to the valence of cues and the valence of contexts. We manipulated the valence of cues and contexts with pictures from the International Affective Picture System. The participants, undergraduate students, showed higher performance when neutral compared to valenced pictures were used for cueing prospective memory. In addition, neutral pictures were more effective as cues when they occurred in a valenced context than in the context of neutral pictures, but the effectiveness of valenced cues did not vary across contexts that differed in valence. The finding of an interaction between cue and context valence indicates that their respective influence on event-based prospective memory task performance cannot be understood in isolation from each other. Our findings are not consistent with by the prevailing view which holds that the scope of attention is broadened and narrowed, respectively, by positively and negatively valenced stimuli. Instead, our findings are more supportive of the recent proposal that the scope of attention is determined by the motivational intensity associated with valenced stimuli. Consistent with this proposal, we speculate that the motivational intensity associated with different retrieval cues determines the scope of attention, that contexts with different valence values determine participants’ task engagement, and that prospective memory task performance is determined jointly by attention scope and task engagement.

## Introduction

Event-based prospective memory, the ability we use for making plans and promises and for carrying them out later in the appropriate context, is affected by the valence of the cues provided for retrieval. A compelling demonstration of this influence was provided by Clark-Foos, Brewer, Marsh, Meeks and Cook [1]. In experiments where subjects were engaged in lexical decision making, the results showed a lower rate of responding when the planned task was cued by means of negatively valenced words (e.g., maggot) than positively valenced words (e.g., puppy).

A few studies have replicated this prospective memory advantage of positively over negatively valenced cues. Specifically, in a study with healthy older adults as well as individuals with depression by Altgassen, Henry, Bürgler and Kliegel [2], the healthy adults’ data showed an advantage for positively over negatively valenced cues; the study also included neutral cues which gave rise to an intermediate level of performance roughly comparable to that obtained with negative cues. The same outcome was reported by Rendell et al. [3] who used the Virtual Week task to explore event-based prospective memory in younger and older adults. A study by Rummel, Hepp, Klein and Silberleitner [4] which examined the influence of valenced cues under different induced mood conditions also revealed an advantage for positive over negative cues in their neutral mood condition, and in addition, it showed a substantially lower performance level when neutral cues were provided for retrieval.

The result of other related investigations are more at odds with the Clark-Foos et al. [1] findings. Altgassen, Phillips, Henry, Rendell and Kliegel [5], in a study with healthy younger and older adults, observed a performance advantage for negatively valenced over positively valenced cues in both age groups. In addition, the younger group showed the same level of performance on neutral and positive cues, while the older group showed much lower performance on the neutral than positive cues. Similarly, a study with younger and older adults by Schnitzspahn, Horn, Bayen and Kliegel [6] showed no overall influence due to cue valence in the younger group, while the older adults showed a lower level of performance with neutral cues than with either positive or negative cues, with the latter two cue types supporting similar levels of performance.

The present study was motivated, in part, by the inconsistent findings from the existing research. We are aware that outcome differences could be due to a number of variables which differed among previous investigations, including participant attributes (e.g., age, mood state), the stimuli used as prospective task cues (e.g., words, pictures), the materials used for the ongoing task, as well as the demands imposed by the ongoing tasks in which the cues appeared. The present study was not intended to investigate the separate and combined influences of all of these variables. Instead, our main aim was to disentangle the effect of two variables that were confounded in some previous studies, namely, the valence of the prospective memory task cues and the valence of the context in which they were presented. As underscored by the foregoing review of previous investigations, most of them have focused on how prospective memory task performance is influenced by cue valence manipulations. To complement this work, we investigated influences due to context valence manipulations as well as the interaction between cue and context valence.

An event-based prospective memory task is a complex activity that affords several different kinds of valence influences. Specifically, the planned task itself may be a positive, negative or neutral activity (cf. Rendell et al. [3]), the cues provided for retrieval could be positive, negative or neutral, and the context in which the cues are presented may be positive, negative or neutral. To the best of our knowledge, task valence was manipulated only in the Rendell et al. [3] study, while the other investigations summarized earlier in this article focused primarily on cue valence influences. In all cases, participants were assigned a neutral prospective memory task (e.g., to press a designated key on the computer keyboard) which had to be performed upon the occurrence of cues that were either neutral or positively or negative valenced. And also in all cases, except for Rendell et al. [3], these cues appeared in the course of an ongoing activity that involved a randomly ordered list of stimuli which were either neutral or positively or negative valenced.

By virtue of using randomly ordered lists composed of neutral stimuli and valenced stimuli for the ongoing tasks, previous investigations were not designed to reveal the independent or distinct influences on prospective memory task performance due to cue valence versus context valence. Moreover, the method used in previous investigations may have obscured the true magnitude of cue valence influences because a cue with a particular valence (e.g., positive) could have been presented either immediately after a neutral stimulus, after a stimulus with the same valence (e.g., positive) or after a stimulus with a difference valence (e.g., negative). To the extent that the cognitive processing elicited by an ongoing-task stimulus endures after its offset, in previous investigations this processing interacted with the activity engaged by the prospective memory task cues, and this confounding might thereby have served to either increase or decrease performance.

The possibility that the true magnitude of cue valence influences might have been obscured in previous studies is suggested by investigations of the electrocortical reactions elicited by valenced and neutral stimuli. By means of event related potentials (ERPs), several studies have shown that valenced stimuli trigger, first, a negative potential that reaches its maximum within about 200 to 300 ms after stimulus onset [7–9], and then a second, longer lasting positive potential [8–10]. The latter potential is sustained for as long as an affective stimulus is presented [8], is still evident even at about 1500 ms after stimulus offset [10], and it has been interpreted as evidence for the selective processing of emotional stimuli, which is assumed to be linked to the activation of the brain’s motivational system [8, 11]. Consistent with such evidence and its interpretation, it is possible that either some type of priming or some type of proactive interference might occur [12–14] when a new stimulus (e.g., a prospective memory cue) is presented while the cognitive system is still engaged with the processing of a previous stimulus; moreover, it seems likely that this type of carry-over will vary depending on the valence and perhaps other properties (e.g., arousal) of two successively presented stimuli. Based on this reasoning, we suspect that the true magnitude of cue valence influences was obscured in previous studies, because in all cases (except for Rendell et al. [3]) the cues in those studies were displayed as part of a randomly ordered list containing stimuli which were either neutral or positively or negative valenced, and stimuli and cues were presented in close temporal proximity to each other.

The present investigation was also motivated by theoretical speculations about the effects of valenced stimuli on the scope of attention. The prevailing view holds that the scope of attention is broadened and narrowed, respectively, by positively and negatively valenced stimuli [15–19]. This view is consistent with the results from flanker task studies [20], more specifically with the finding of larger flanker interference effects after exposure to positively than negatively valenced stimuli [21–22]. Positive affect is known also to influence higher-level cognition. For example, it increases the range of items accepted as members in a categorization task [23], and the unusualness of items generated on a word association task [24]. Therefore, consistent with such findings, it is plausible that subjects consider more potential interpretations of positively than negatively valenced cues, or that they are more likely to consider the prospective task relevance of cues appearing in a positively than negatively valenced context and that this processing difference translates into higher prospective memory task performance.

To explore the influence of context and cue valence on event-based prospective memory task performance, the present research employed pictures from the International Affective Picture System (IAPS) [25]. We selected the pictures so as to differ in valence while having similar arousal ratings. The pictures were presented in blocks, each with 25 pictures from the same valence bin (i.e., positive, neutral or negative), followed by one picture—a prospective memory task cue—from either the same or a different valence bin. Participants were assigned two activities, a ‘spot-the-differences’ task and an affectively neutral event-based prospective memory task. For the latter task, participants were told to press a designated key on the computer keyboard upon encountering a picture belonging to a specified category (e.g., a furniture item). For the spot-the-differences task, participants were required to count perceptual differences between two copies of the same picture displayed side by side. By employing sequences of pictures with the same valence, our method created a distinct valence context for each prospective memory task cue, and thereby permitted the separate assessment of context and cue valence influences. We made the assumption that a longish sequence of stimuli from the same valence category might strengthen the valence influence on attention scope [13, 26–27], thereby accentuating the performance advantage of the positive over negatively valence condition.

## Experiment 1

The main aim of Experiment 1 was to generalize previous investigations concerned with cue valence by focusing on the effects produced by context valence manipulations.

### Method

**Participants & Design.** Seventy-one undergraduate student volunteers participated in this experiment in return for course credit. The experiment had valence context condition (positive, neutral and negative) as a within subject factor.

**Instruments.** In the course of the experiment, each participant was administered two standardized instruments, the 60-item NEO Five Factor Personality Inventory [28], and the Digit Symbol Substitution Test [29]. Analyses of the results from these instruments showed no significant influences and thus are not reported.

**Stimuli**. We used color pictures from the IAPS [25] selected according to their valence and arousal ratings. We sampled the collection until we had 50 pictures for each valence context condition, respectively, with positive (M = 7.06), neutral (M = 4.96) and negative (M = 2.80) valence ratings, that were similar in terms of their arousal ratings (M = 4.74, 95% CI: 4.47 to 5.01; M = 4.70, 95% CI: 4.38 to 5.02 & M = 5.00, 95% CI: 4.67 to 5.33, respectively, for the pictures in the positive, neutral and negative valence condition). These pictures showed a variety of humans and human interactions (e.g., a couple kissing; a child crying at the dentist), scenes of built and natural environments (e.g., a cityscape; sunset over the mountains) and scenes with animals (e.g., a kitten; a horse jumping; a snake pit).

We selected an additional six neutral/medium valence, medium arousal pictures from the same source and according to the same general criteria. These pictures were used in connection with instructions and for practicing the spot-the-differences task. In addition, we required six pictures with the same attributes (i.e., neutral/medium valence, medium arousal) for use as prospective memory task cues, with each of them depicting a familiar item of furniture (e.g., a table, a couch). However, because the IAPS includes only one picture of a familiar furniture item, we selected the prospective cue pictures from a variety of Internet sources.

For the spot-the-differences task, we created eight transparent visual masks that could be displayed over the IAPS pictures. Each mask consisted of a 4 by 5 regularly spaced invisible line grid, with colored round dots marking each line intersection (i.e., each mask showed a total of 20 colored dots). On each mask, most of the dots were rendered in bright red, but a randomly selected two or three of them were shown in blue, green or purple. When displayed on a 640 x 480 pixel monitor in the course of the experiment, each dot had a diameter of about 4 millimeters.

**Procedure.** The main parts of the experiment were carried out with a desktop computer equipped with a 23 inch LCD monitor and running E-Prime V2.0 [30]. All participants were tested individually in a session lasting about one hour. The experiment was conducted with the approval of the University of British Columbia Research Ethics Board.

After giving written consent, participants were instructed about the prospective memory task. Specifically, they were told that “if ever you see a picture of any furniture item in the course of the experiment, stop whatever you are doing and immediately press the Q-key on the computer keyboard”. To ensure comprehension of these instructions, the experimenter showed a printed picture of a furniture item, clearly identified the Q-key on the keyboard and participants were required to repeat the task instructions. Immediately after these instructions, each participant completed a paper-and-pencil version of the DSST and NEO-FFI according to the standardised instructions for these instruments, and then—about 15 minutes after receiving the prospective memory task instructions—they proceeded to the spot-the-differences task.

**Fig 1:**
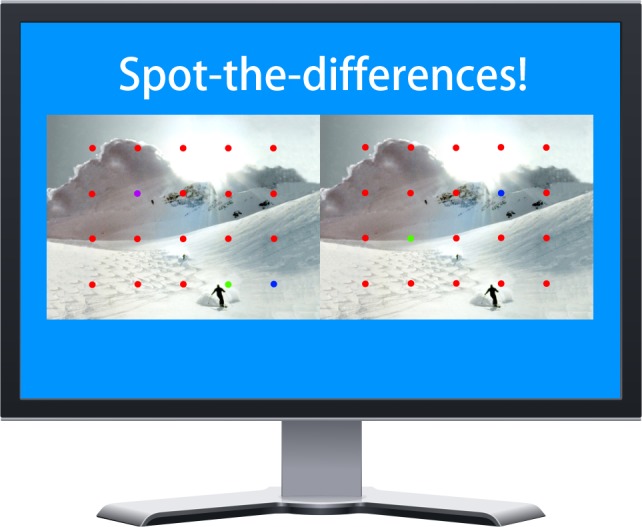
Spot-the-differences task display. The spot-the-differences task required counting the number of dots that differed between two copies of a picture (i.e., 5 in the illustration).

The spot-the-differences task consisted of a series of trials. For each trial, the computer displayed two copies of the same picture, side by side, and overlaid on each picture appeared one of the transparent visual masks, as shown in Fig. 1. The selection of the visual masks was random, and as a consequence, and because each mask had two or three non-red dots, the total number of dots that were different between each mask pair could be as low as four and as high as six. Participants were instructed to inspect each picture pair, to count the number of differences between them, and to enter this count on the keyboard number pad. When a difference count was entered, the current display disappeared and after 750 ms was replaced with a new display. In addition, each incorrect count entry triggered a 250 ms sound to remind participants about the importance of counting correctly.

Each participant received six practice trials on the spot-the-differences task, was reminded that both speed and accuracy would be recorded, and then completed six blocks each with 26 trials. On the last trial of each block, one of the prospective memory cues, selected randomly and without replacement, was displayed in the same manner as described in the preceding paragraph. On each of the initial 25 trials of each block, one of the selected IAPS picture was displayed selected randomly and without replacement from one of the three valence bins. By this arrangement, we created blocks in which a neutral valence prospective cue would be displayed after, respectively, a series of 25 positive, 25 neutral or 25 negative valence IAPS pictures. The six blocks administered to each participant were arranged into three sets of two blocks, where the two blocks in each set were always from the same context valence condition, and the order of the two blocks was randomly determined for each participant; the order of the sets was balanced by means of a Latin Square, with participants successively assigned to the possible set-orders according to their appearance in the lab. The entire series of 156 trials was delivered without interruption, according to each participant’s self-selected pacing, and was completed in about 20 minutes. Immediately after this phase, participants were interviewed about the experiment and questioned to ascertain their memory for the prospective task instructions.

### Results

The main dependent variables were accuracy and speed on the prospective memory task as well as accuracy and speed on the spot-the-differences task. The alpha level was set at .05 for all statistical tests.

**Fig 2:**
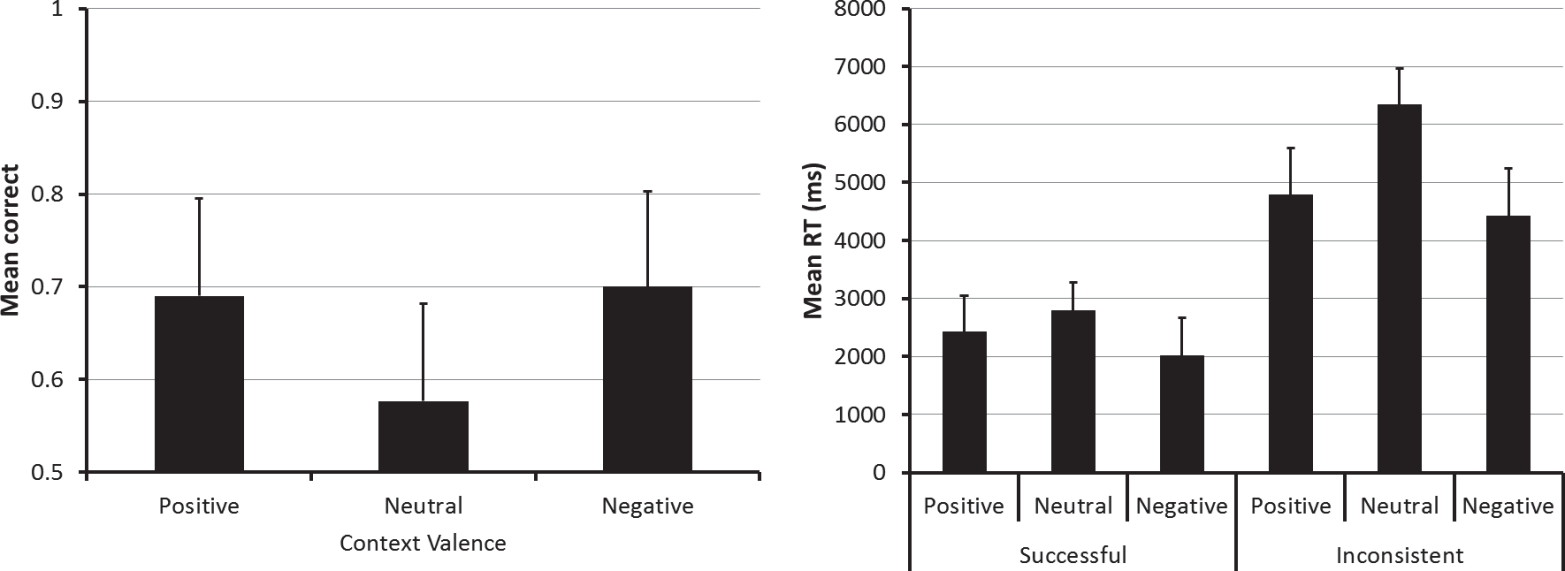
Prospective memory task performance in Experiment 1. The figure shows prospective memory task accuracy (left panel) and speed (right panel) as a function of the valence of the context (positive, neutral and negative) in which the retrieval cues were displayed. The error bars represent the 95% confidence interval for each mean.

On the prospective memory task, the accuracy data, in the left panel of Fig. 2, showed that subjects were more successful with cues presented in the context of positively and negatively valenced pictures than in the context of neutral pictures. An ANOVA of the accuracy data showed a significant effect due to context valence, *F* (2, 140) = 3.95, *p* = .021, ηp^2^ = .053. [Not that for ηp^2^ values, 0.01, 0.06 and 0.14 are considered small, medium and large effect sizes, respectively [31].] Follow-up t-tests showed that in the neutral valence context condition, accuracy was significantly lower than in the positive valence condition, *t* (70) = 2.33, *p* = .023, or in the negative valence condition, *t* (70) = 2.32, *p* = .023. Accuracy was not significantly different between the positive and negative valence conditions, *t* (70) = .373.

To learn about possible strategies participants’ might have used, we examined the speed of responding to the prospective memory task cues. For this purpose, we created two groups, one consisting of 44 subjects—herein called the successful responders—who made at least one correct prospective task response in each context valence condition, and the other consisting of those 27 subjects—herein called the inconsistent responders—who failed to make a prospective task response in at least one of these conditions. For the first and more relevant group, we used only the speed data from correct responses to the prospective task cues. For the second group, and in order to enable an easy statistical comparison between successful and inconsistent responders, we included the speed data from all responses to the prospective task cues (i.e., independent of whether they were prospective task responses or spot-the-difference task responses). An ANOVA of the response speed data, in the right panel of Fig. 2, showed a significant main effect due to context valence condition, *F* (2, 138) = 12.87, *p* < .01, ηp^2^ = .157, as well as due to response group, *F* (1, 69) = 57.20, *p* < .01, ηp^2^ = .453. It also showed an interaction between context valence condition and response group, *F* (2, 138) = 2.98, *p* = .054, ηp^2^ = .041, that was just short of significance.

**Fig 3:**
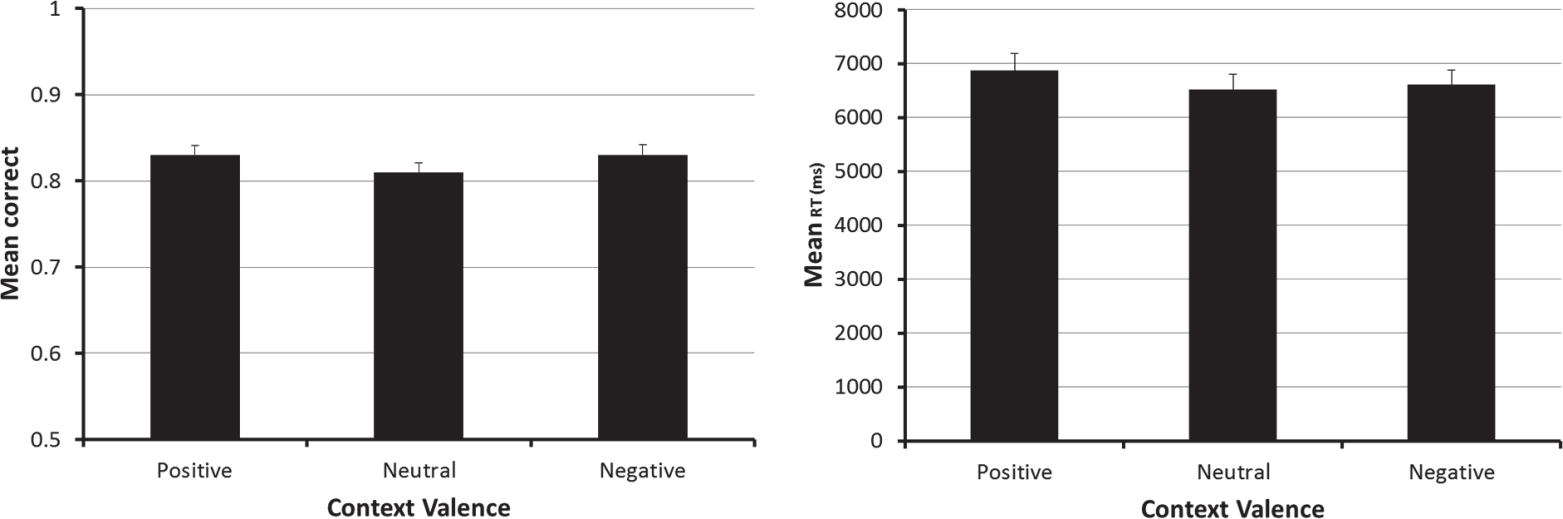
Spot-the-differences task performance in Experiment 1. The figure shows spot-the-differences task accuracy (left panel) and speed (right panel) as a function of the valence of the pictures used for creating the positive, neutral and negative context conditions. The error bars represent the 95% confidence interval for each mean.

We also examined the accuracy and speed of responding on the spot-the-differences task for possible influences due to the context valence manipulation. The left panel of Fig. 3 shows that spot-the-differences task accuracy was similar across all three valence conditions. This observation was confirmed by an ANOVA which revealed that the small differences between the means failed to achieve significance, F (2, 140) = 2.58, p = .079, ηp^2^ = .036. Nevertheless, in view of the small differences in accuracy, we used data from only the correct spot-the-difference task trials for the analysis of performance speed. The means of subjects’ median response speeds from these trials, shown in the right panel of Fig. 3, also were not significantly different from each other, F (2, 140) = 1.62, p = .20, ηp^2^ = .023.

The absence of a context valence influence on either spot-the-differences task accuracy or speed suggests that this variable did not have a selective influence on participants’ responses to the prospective memory task cues. Moreover, a comparison of the speed data in Figs. 2 and 3 indicates that the spot-the-differences task was more time-consuming than the prospective memory task, that correct prospective memory task responses required only about 50% of the time needed for making spot-the-differences task responses. This latter finding is important for at least two reasons. First, it provides prima-facie evidence that participants accessed the content of the pictures used for the spot-the-differences task. Second, it suggests that the failure to find a context valence influence on the spot-the-difference task accuracy and speed occurred despite participants’ processing of the content of the stimulus pictures.

A final analysis examined the possibility that in the course of the experiment participants’ became aware of the regular occurrence of prospective task cues and this awareness influenced how they carried out the assigned tasks. To address this possibility, we examined changes in performance across the six prospective task cues presented to each participant, changes in performance across the six blocks of the spot-the-differences task, and the observations made by participants in the post-experiment interview. In the post-experiment interview none of the participants volunteered information about to the regular occurrence of the prospective task cues, although many mentioned becoming more proficient on the spot-the-differences task. Performance on the prospective cues showed an increase in correct responding from the first cue (M = .48) to the sixth cue (M = .77), F (5, 350) = 8.41, *p* < .01, ηp^2^ = .107. This finding is familiar from previous studies [32], and is assumed to occur because each successful response to a cue is a powerful reminder of the prospective memory task. On the spot-the-difference task performance accuracy was the same across the six blocks (1^st^ Block M = .82; 6^th^ Block M = .81), F (5, 350) = 1.44 p = .21, but the time required for making correct spot-the-differences task responses decreased substantially across blocks (1^st^ Block M = 7632 ms; 6^th^ Block M = 5779 ms), F (5, 350) = 32.87, p < .01, ηp^2^ = .32. This latter finding rules out the possibility that participants allocated less attention to the spot-the-differences task and switched to cue monitoring as they progressed through the experiment.

### Discussion

Experiment 1 examined context valence influences on event-based prospective memory task performance and it produced two novel findings: first, higher performance when neutral cues were presented in either a positively or negatively valenced context compared to a neutral context, and second, no difference in performance levels between the positively and negatively valenced context conditions. This pattern of performance is different from that observed in previous investigations, most of which have focused on the valence of the cues provided for prospective memory retrieval, thereby suggesting that prospective memory task performance is affected differently by context-valence versus cue-valence manipulations.

Experiment 1 is a conceptual replication of Experiment 2 by Clark-Foos et al. [1] in which prospective memory retrieval was cued with neutral words embedded in sentences that were affectively neutral or either positively or negatively valenced. By contrast to our results, however, Clark-Foos et al. reported higher performance with cues inserted into positively than negatively valenced sentences, and the highest level of performance with cues embedded in neutral sentences. We are unable to explain the difference in outcomes other than to suggest that they stem from differences in the methods used for creating the neutral and valenced contexts (i.e., sentences versus picture sequences). In addition, because there exists no method for ascertaining and comparing the strength of the valence manipulation used by Clark-Foos et al. versus in Experiment 1, we are unable to rule out an explanation of the outcome differences which invokes valence strength or level and its supposed effects on the scope of attention.

The results from Experiment 1 appear to be inconsistent with the theoretical claim that the spotlight of attention is broadened and narrowed, respectively, by positively and negatively valenced stimuli [15–19]. Consistent with this claim, we anticipated that subjects would consider more potential interpretations of cues embedded in positively than negatively valenced contexts, that they would be more likely to consider the cues’ prospective memory task relevance and that this processing difference would translate into higher performance. By contrast to this expectation, Experiment 1 showed no performance difference between the positive and negative context conditions.

Our failure to find a prospective memory performance difference between the positive and negative context condition may not be surprising, however, in view of the affectively neutral pictures we used as cues in all conditions of Experiment 1. We expected a performance advantage in the positive versus negative valence condition on the assumption that the scope of attention is determined by the context pictures and that it would carry over to the processing of each cue positioned immediately after each picture series. We made this assumption in view of ERP research showing that electrocortical potentials are sustained for as long as an affective stimulus is displayed [8] and are evident even at about 1500 ms after stimulus offset [10]. However, we do not know how the electrocortical activity induced by an affective stimulus is altered by the occurrence of a new, affectively neutral stimulus. For this reason, we must consider the possibility that the scope of attention does not carry-over from one stimulus to the next, or that it can change very quickly and is determined entirely by the stimulus currently being processed. If attention scope is controlled in this manner, our finding of no performance difference between the positive and negative valence conditions is not surprising.

Even if we accept the assumption that prospective memory task performance is determined entirely or primarily by cue-induced processing (i.e., not influenced in any way by context-induced processing), we still need to explain the finding of lower performance in the neutral context condition than in the valenced context conditions. However, before considering theoretical accounts for this finding, we must rule out a less interesting alternative, namely, the possibility that this finding is nothing more than a kind of contrast effect. That is, it might be argued that because of the valence difference between the cue and context pictures, neutral cue pictures are more effective and more likely to be noticed as cues when presented in the context of either negatively or positively valenced pictures compared to neutral pictures. To examine the possibility, we conducted a detailed post-experiment interview with each participant and none of them made any comment that would support a contrast effect account. Nevertheless, Experiment 2 was designed to rule out this type of account and to differentiate between it and the more interesting alternatives interpretations suggested by theoretical assumptions about how valenced contexts and cues might affect the scope of attention.

## Experiment 2

By focusing on how context valence influences event-based prospective memory task performance, Experiment 1 complements previous investigations that examined prospective task effects due to the valence of the cues provided for retrieval. Experiment 2 was designed to replicate Experiment 1, and to investigate the separate influence as well as the combined influences due to both context valence and cue valence. The basic method of Experiment 2 was the same as that used for Experiment 1, except with cue valence added as a factor. In the neutral cue condition of Experiment 2, neutral pictures were used for cuing prospective memory retrieval, while positively and negatively valenced pictures respectively were used in the positive and negative cue conditions. By this manner of combining context and cue valence in Experiment 2, we created three conditions in which the context and cue were from the same valence bin, and six conditions in which pictures from different valence bins were used as context and cue stimuli. This method permitted us to examine the pure contrast explanation that is a possible account for the lower performance in the neutral context condition of Experiment 1. Consistent with the pure contrast explanation, we expected higher prospective memory task performance in the six conditions where pictures with different valences were used as cues and for creating contexts, compared to the three conditions where pictures from the same valence bin were used as cue and context stimuli.

### Method

**Participants & Design.** One hundred and ninety-five undergraduate student volunteers from the University of British Columbia participated in this experiment in return for course credit. The experiment had context valence (positive, neutral & negative) as a within-subject factor, and cue valence (positive, neutral and negative) as a between-subjects factor. Sixty-five subjects, respectively, were randomly assigned to the positive, neutral and negative cue valence conditions.

**Visual Stimuli**. We used the IAPS pictures from Experiment 1 for manipulating context valence, as well as for practicing the spot-the-differences task, and we used the grid masks from Experiment 1 for implementing the spot-the-differences task. In addition, for use as prospective memory task cues, we selected three additional sets with six pictures each, from the same source and according to the general method described in Experiment 1. Specifically, one of these cue sets consisted of positively valence items (M = 7.31), depicting puppies and dogs, a second consisted of neutral valence items (M = 5.27), depicting different types of mushrooms, and the third consisted of negatively valence items (M = 2.32), depicting different types of car crashes. The selected pictures were similar in terms of their arousal ratings (M = 4.04, 95% CI: 3.67 to 4.41; M = 3.77, 95% CI: 3.36 to 4.18; M = 3.56, 95% CI: 2.92 to 4.19, respectively for the pictures in the positive, neutral and negative valence condition).

**Procedure.** All parts of the procedure were the same as for Experiment 1 except for the instructions for the prospective memory task which varied according to the cue valence condition. As in Experiment 1, the prospective memory task instructions were given immediately after obtaining written consent, and participants in the positive valence cue condition were told that “if ever you see a picture of *a puppy*, *a dog or dogs* in the course of the experiment, stop whatever you are doing and immediately press the Q-key on the computer keyboard”. In the neutral valence cue condition, the words in italics were replaced with the phrase *a mushroom or mushrooms*, and in the negative valence cue condition, these words were replaced with the phrase *a car crash*. To ensure comprehension of the instructions, the experimenter identified the Q-key on the keyboard and participants were required to repeat the task instructions. Immediately after these instructions, each participant completed a paper-and-pencil version of the DSST and NEO-FFI according to the standardised instructions for these instruments, and then—about 15 minutes after the prospective memory task instructions—they proceeded to the spot-the-differences task, which was also administered in the exact same manner as in Experiment 1.

### Results

The main dependent variables were accuracy and speed on the prospective memory task as well as accuracy and speed on the spot-the-differences task in each experimental condition.

**Fig 4:**
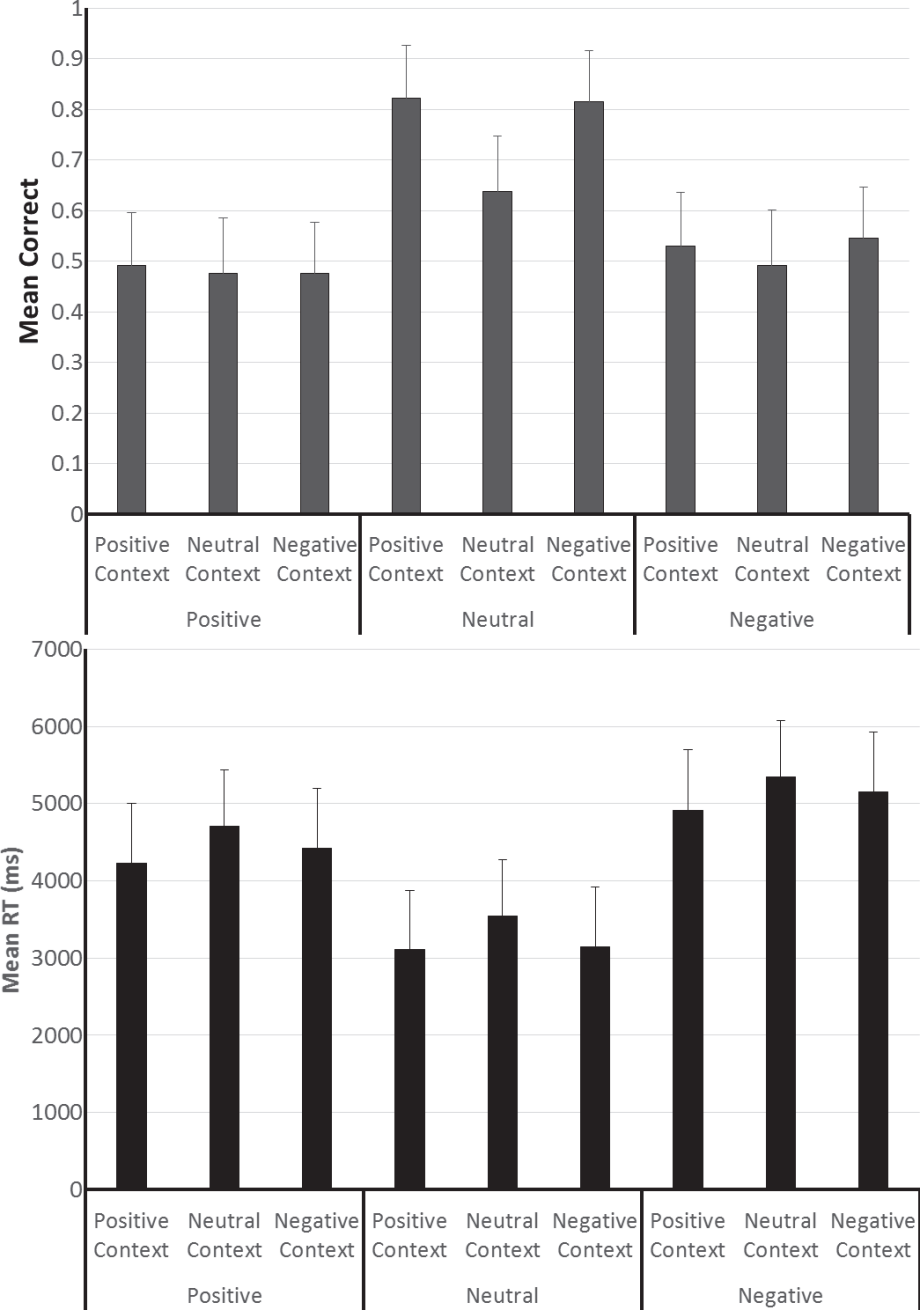
Prospective memory task performance in Experiment 2. The figure shows prospective memory task accuracy (top panel) and speed (bottom panel) as a function of the valence of the pictures used as prospective memory task cues, and the valence of the pictures used for creating the positive, neutral and negative context conditions. The error bars represent the 95% confidence interval for each mean.

On the prospective memory task, the accuracy data in the top panel of Fig. 4 showed a higher level of performance when retrieval was cued with neutral pictures, than with either positively or negatively valenced pictures. In addition, the results showed an influence due to the valence of the context but only when neutral cues were used; there was no influence due to context valence with either the positively or negatively valenced cues. This summary of the findings was supported by the results of an ANOVA which showed a significant main effect due to cue valence, *F* (1, 192) = 10.49, *p* < 01, ηp^2^ = .099, and due to context valence, *F* (2, 384) = 5.92, *p* < .01, ηp^2^ = .03. The interaction between these factors was also significant, *F* (4, 384) = 2.73, *p* = .029, ηp^2^ = .028. Follow-up analyses with the data from each cue-type condition revealed a significant main effect due to context valence for neutral cues, *F* (2, 128) = 7.95, *p* < .01, ηp^2^ = .11, but neither for positively nor negatively valenced cues.

For the purpose of examining the speed of responding to the prospective memory cues, it was difficult to partition and analyze the data as in Experiment 1, and for this reason, we focused on the speed data from all responses to the prospective memory cues. The mean response speed data summarized in the bottom panel of Fig. 4 showed that participants were faster to response to neutral cues than to either positively or negatively valenced cues. An ANOVA of the response speed data showed a significant main effect due to cue type, *F* (2, 192) = 8.14, *p* < 01, ηp^2^ = .078. No other main or interaction effects were significant.

The finding that participants were faster to respond to neutral cues than to either positively or negatively valenced cues is consistent with the outcome of Experiment 1. In that experiment, when a cue was displayed, participants were substantially faster to make a prospective task response than a correct spot-the-difference task response. Because participants in Experiment 2 were over 20% more likely to make a prospective response to neutral cues compared to either the positively or negatively valenced cues, it is likely that this difference accounts for the overall speed differences highlighted by the data in the bottom panel of Fig. 4.

**Fig 5:**
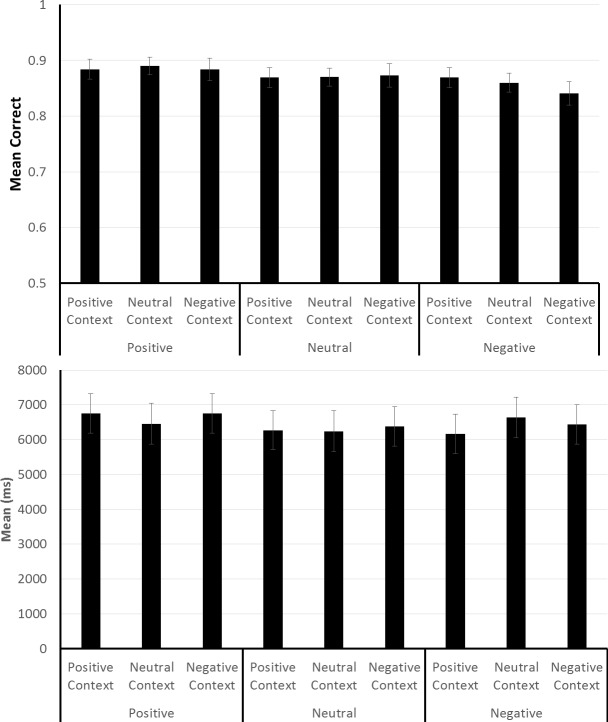
Spot-the-differences task performance in Experiment 2. The figure shows spot-the-differences task accuracy (top panel) and speed (bottom panel) as a function of the valence of the pictures used as prospective memory task cues, and the valence of the pictures used for creating the positive, neutral and negative context conditions. The error bars represent the 95% confidence interval for each mean.

On the spot-the-differences task, accuracy varied minimally across the cue and context valence conditions, as highlighted by the means in the top panel of Fig. 5. This observation was borne out by an ANOVA which showed a significant effect due to cue type, F (2, 192) = 3.34, p = .038, ηp^2^ = .034, as well as an interaction between cue type and context valence, F (4, 384) = 2.73, p = .029, ηp^2^ = .028. Although not anticipated, the observed small differences in accuracy were more prominent in the conditions with the negatively valence cues compared to the conditions with either neutral cues or positively valenced cues. Overall spot-the-differences task accuracy was about 5% higher in Experiment 2 than in Experiment 1, and this difference is larger than any of the mean differences observed within Experiment 2. In view of the small differences in accuracy, we used data from only the correct spot-the-difference task trials for the analysis of performance speed. The means of subjects’ median response speeds from these trials are shown in the bottom panel of Fig. 5. An analysis of the response speeds showed no significant main or interaction effects.

### Discussion

The new findings from Experiment 2 are, first, the higher level of prospective memory task performance when retrieval was cued with neutral pictures, compared to either positively or negatively valenced pictures, and second, a performance influence due to context valence but only when neutral cues were provided for retrieval. The effect due to context valence in the neutral cue conditions of Experiment 2 replicates the results from Experiment 1. In these conditions, performance was about 10% higher across the board in Experiment 2 than 1, and this difference might be due either to the different pictures used as cues (i.e., pictures of mushrooms in Experiment 2 versus pictures of furniture in Experiment 1) or more likely due to differences in the participant samples.

The finding that context valence influenced prospective memory task performance only when neutral cues were provided for retrieval, and not when either positively or negatively valence cues were used, rules out the pure contrast explanation considered in connection with the results from Experiment 1. By a pure contrast account, we had expected a higher level of performance in all six conditions with a valence difference between the context and cue pictures, compared to the three conditions where context and cue pictures were selected from the same valence bin. The data in Fig. 4 are not consistent with this expectation.

The present study was inspired by the prevailing theoretical view which holds that the scope of attention is broadened and narrowed, respectively, by positively and negatively valenced stimuli [15–19]. This view does not specify whether the scope of attention is modulated by the valence of pictures provided as cues or by the valence of the contexts. However, it predicts a difference in the outcome for pictures from different valence bins. Specifically, on the assumption that a broadening of attention causes a more in-depth examination of stimuli and their affordances, we expected that prospective memory task performance would be higher with positive cues than neutral cues, with the reverse performance pattern for negative over neutral cues. The results are not consistent with this general expectation: They show no evidence of a performance advantage for positive over negative cues; they also show no evidence of a performance advantage of positive contexts over negative contexts.

The finding that prospective memory performance was influenced by context valence only when the task was cued with neutral as opposed to valenced pictures points to a complex interaction between cue and context valence. This interaction also cannot be explained by the prevailing theoretical view even if we make the additional assumption that cue and context valence have an additive effect on the scope of attention. By the assumption that prospective memory performance is positively related to the scope of attention, and by the stipulation that attention scope is influenced similarly by both cue and context valence, performance should have been highest in the condition where positively valenced cues were presented in a positively valenced context. By contrast to this expectation, we found the highest level of performance when neutral cues were presented in valenced contexts.

Harmon-Jones, Gable and Price [33] and Huntsinger [3] recently have challenged the prevailing view on how valenced stimuli affect the scope of attention. They argued that many previous investigations compared the effects of negatively valenced stimuli of high approach motivational intensity with positively valenced stimuli of low approach motivational intensity, and suggested that this confounding of valence and motivational intensity is the cause of the observed narrowing and broadening of the scope of attention [33, 35]. Harmon-Jones, Gable and Price [33] defined approach motivational intensity as “the strength of urge to move toward/away from a stimulus” (p. 301). The present experiments were not designed to contrast this theoretical proposal with the prevailing view. However, the positively and negatively valenced pictures we used for our experiments were selected so as to be about equally distant in the IAPS norms from the neutral pictures. Therefore, if the normative IAPS valence ratings can be used as a proxy for approach motivational intensity, we might have anticipated the same prospective memory effect for positively and negatively valenced cues, and perhaps also for positive and negative contexts.

The proposal that attention scope is related to approach motivational intensity does not specify the levels of motivational intensity that are associated with the broadening or narrowing of the scope of attention, and for this reason there is no basis for predicting whether prospective memory task performance in our valenced conditions should have been higher or lower than performance in the neutral conditions. However, the finding that in Experiment 2 overall prospective memory task performance was lower with valenced cues than with neutral cues is consistent with the notion that our valenced cues narrowed the scope of attention. It might be argued that because the scope of attention was narrowed, positively and negatively valenced cues were examined for fewer potential affordances than neutral cues, and as a consequence, participants were less likely to respond to the prospective task relevance of the former cues. Future research will examine this possible interpretation.

The proposal that attention scope is related to approach motivational intensity also fails to account for the finding in Fig. 4 that prospective memory performance was influenced by context valence only when the task was cued with neutral as opposed to valenced pictures. To explain this outcome, we speculate that cue and context valence exert different kinds of influences on prospective memory task performance. Specifically, we propose that cue valence determines the breadth of processing that will occur, and consistent with speculations in the foregoing paragraphs, we assume that our participants were more likely to consider the affordances of the neutral cues compared to the valenced cues. In addition, we speculate that the context in which a cue is presented might drive or energize processing, that participants are more animated or engaged by valenced contexts than neutral context [1]. Consistent with these two assumptions, it follows that prospective memory performance should be higher when neutral cues (i.e., cues that engage relatively broad processing) occur in the context of valenced stimuli than in the context of neutral stimuli. However, with valenced cues which induce a relatively narrow type of processing, the additional processing energy provided by valenced contexts is unable to make a positive difference to prospective memory task performance.

The proposal that cues determine the scope of attention while contexts serve to energize processing speaks to the question we raised in the Discussion of Experiment 1, that is, whether or not the scope of attention is determined jointly by cue valence and context valence. By our interpretation, the findings from Experiment 2 suggest that the scope of attention is determined primarily by cue valence (i.e., the approach motivational intensity created by cues), and that the only carry-over from contexts to cues is in the form of processing energy. Although speculative, this proposal is consistent with previous research which has shown that the electrocortical activity elicited by a valenced stimulus is evident even at about 1500 ms after stimulus offset [8, 10], and more specifically with the suggestion that this post-stimulus activity is due to the activation of the brain’s motivational system [8, 11]. To our knowledge, previous research has not explored how this type of post-stimulus activity is affected by the encounter with a new stimulus. Moreover, future research will have to determine whether our findings will generalize to situations where, for example, the temporal separation between cues and context is larger than in our experiments or where weaker context manipulations are used.

The findings from our experiments show both similarities and differences with the outcome of previous investigations, most of which focused on cue valence. Specifically, our finding that overall prospective memory task performance was higher with neutral cues than with either positively or negatively valenced cues is consistent with the outcome of the seminal work by Clark-Foos et al. [1], but in contrast to their study, we failed to find the performance advantage for positively over negatively valenced cues. In addition, our finding of similar levels of prospective memory task performance for positively and negatively valenced cues replicates and extends the outcome reported by Schnitzspahn et al. [6], but by contrast to their study, we found a higher level of performance with neutral cues, whereas their study revealed, for older adults, a lower level of performance with neutral cues, and for younger adults, the same level of performance with all cue types. As explained in the Introduction, such outcome differences could be due to a number of variables, including participant attributes (e.g., age, their cognitive/emotional state), the stimuli used as prospective task cues (e.g., words, pictures) and the stimuli used for the ongoing tasks. In addition to these factors, it is also possible that differences arose because of the manner in which cue and context valence were deliberately confounded in previous studies as opposed to being systematically manipulated as in the present study. The present study was not designed to investigate such potentially confounding factors. Instead, our goal was to identify the separate and interactive influences due to cue valence and context valence. Our finding that cue and context valence jointly influence prospective memory task performance provides a new motivation for research and a more systematic exploration of the valence factors that determine event-based prospective memory task performance.

## Conclusion

To augment the extant research on the influence of valenced stimuli on event-based prospective memory task performance, we used valenced and non-valenced pictures as cues and displayed them in valenced and non-valenced contexts. The results showed higher event-based prospective memory task performance when it was cued by means of neutral pictures compared to either positively or negatively valenced pictures. It also revealed that neutral pictures were more effective as cues when they occurred in a valenced context than in a neutral context, and that the effectiveness of valenced picture cues did not vary across differently valenced context conditions. The occurrence of an interaction between cue valence and context valence indicates that their respective influence on event-based prospective memory task performance cannot be understood in isolation from each other. Our findings cannot be accommodated by the prevailing view which holds that the scope of attention is broadened and narrowed, respectively, by positively and negatively valenced stimuli. Instead, our findings are more supportive of the recent alternative proposal from Harmon-Jones, Gable and Price [33] and Huntsinger [34] that the scope of attention is determined by the approach motivational intensity triggered by valenced stimuli. Consistent with this account, we propose that the motivational intensity of cues provided for retrieval determines the scope of attention, and that context with different valence values determine participants’ level of engagement.

## Supporting Information

S1 Table Valence 1 Complete Data Set. (XLSX)

S2 Table Valence 2 Complete Data Set. (XLSX)
